# Improving Health Care Quality for Older Adults in Sub-Saharan Africa: Lessons for Global Health Systems

**DOI:** 10.1093/geroni/igaf122.4141

**Published:** 2025-12-31

**Authors:** Judith Ani

**Affiliations:** Walter Sisulu University, Mthatha, Eastern Cape, South Africa

## Abstract

This study examined how older adults in a Sub-Saharan African community experience healthcare interactions and navigate health and disability challenges, with an emphasis on identifying system-level gaps and opportunities for quality improvement in healthcare delivery. Using a qualitative design, 36 semi-structured interviews were conducted with adults aged 65 years and older, recruited purposively to capture diverse household and social contexts. Data were thematically analyzed to explore lived experiences of care access, quality, and coping strategies. Participants highlighted three recurring challenges in the quality of care: inadequate provider responsiveness to age-related conditions, structural inequities such as long travel distances and out-of-pocket costs, and lack of culturally sensitive communication in clinical encounters. In response, older adults adopted adaptive coping strategies (community caregiving networks, spirituality, and alternative medicine use) and maladaptive strategies (delaying or avoiding care). While these strategies mitigated immediate hardship, they often undermined long-term health outcomes. The findings underscore the urgent need for quality improvement innovations tailored to older populations in low-resource settings. Strengthening provider training in geriatric competencies, integrating community health workers into formal systems, and implementing financial protection measures can enhance care equity. These insights from Sub-Saharan Africa offer globally relevant lessons for advancing age-friendly, patient-centered care in diverse health systems.

